# Metabolic syndrome in patients with lichen planus: A case‐control study

**DOI:** 10.1002/ski2.315

**Published:** 2023-11-30

**Authors:** Mahesh Mathur, Neha Thakur, Sunil Jaiswal, Gautam Das, Swati Shah, Srijana Maharjan, Supriya Paudel, Anjali Shrestha, Hari Prasad Upadhyay

**Affiliations:** ^1^ Department of Dermatology, Venereology and Leprology College of Medical Sciences and Teaching Hospital Bharatpur Nepal; ^2^ Department of Community Medicine College of Medical Sciences and Teaching Hospital Bharatpur Nepal

## Abstract

**Background:**

Lichen Planus (LP) is a chronic dermatosis affecting the skin and mucous membranes. Chronic inflammation and oxidative stress in patients with LP is a trigger predisposing to Metabolic Syndrome.

**Objectives:**

To study the association of Metabolic Syndrome in patients with LP.

**Materials and Methods:**

A hospital‐based prospective case‐control study was conducted from April 2021 to January 2023 including 75 histopathologically confirmed patients with LP and 82 age and sex‐matched controls according to the inclusion and exclusion criteria. Metabolic Syndrome was diagnosed using Modified National Cholesterol Education Programme Adult Treatment Panel III criteria. Statistical analysis of the data was performed using Statistical Package for the Social Sciences software, version 26. The chi‐square test was used for data analysis.

**Results:**

The majority (30.6%) of the patients belonged to the age group 31–40 years. The mean age of patients with LP was 46.13 ± 14.9 years. Female predominance (69.3%) was observed in our study. Patients with classic LP (54.6%) were predominantly observed. Metabolic Syndrome was significantly prevalent in LP patients than in controls (32% vs. 13.4%, *p* = 0.005, OR 3.037) and was significantly associated with morphology (only oral mucosal involvement, 61.5%, *p* 0.027, OR 3.9), severity (severe LP, 58.6%, *p* < 0.001, OR 7.79), and duration of the disease (≥6 months, 55.5%, *p* 0.001, OR 5.42). 71% of Metabolic Syndrome was observed in females (*p* 0.847). Among patients with metabolic syndrome, the majority belonged to the age group between 31 and 40 years (37.5%, *p* 0.378). Systolic and Diastolic Blood Pressure values (≥130/85 mm of Hg), Serum Triglycerides (≥150 mg/dl), and Low‐Density Lipoprotein (>130 mg/dl) were significantly elevated, and High‐Density Lipoprotein (<40 mg/dl) was significantly low in LP than in controls (*p* < 0.05).

**Conclusion:**

The study showed a significant association of Metabolic Syndrome in patients with LP. Thus, patients with LP need to be screened to avoid complications associated with Metabolic Syndrome that is, Diabetes Mellitus, Cardiovascular Disease, colorectal cancer, and stroke.

## Graphical abstract

Chronic inflammation and oxidative stress in patients with LP is a trigger predisposing to Metabolic Syndrome. Our study showed a significant association of Metabolic Syndrome in patients with Lichen Planus. Thus, patients with Lichen Planus need to be screened to avoid complications associated with Metabolic Syndrome.

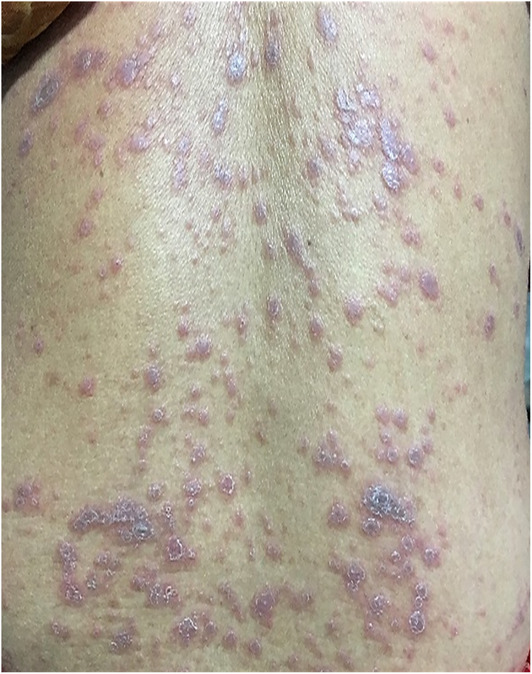

1

1 **What is already known about this topic?**

- Lichen Planus (LP) is a chronic inflammatory autoimmune dermatosis that affects the skin, hair, nails, and mucous membranes which is mediated predominantly by CD8+ T cells. Recent evidence suggests that chronic inflammation and oxidative stress in dermatological disorders contribute to a higher risk for Metabolic Syndrome however, there is a paucity of literature showing a significant association of Metabolic Syndrome in LP.

**What does this study add?**

- Our study showed a significant association of Metabolic Syndrome in patients with LP suggesting the need for screening and early therapeutic intervention to prevent complications of Metabolic Syndrome that is, Diabetes Mellitus, Cardiovascular disorders, colorectal cancer, and stroke.

## 2 INTRODUCTION

Lichen Planus is a chronic inflammatory dermatosis affecting the skin, mucous membranes, hair, and nails.^1^ The reported prevalence of LP is around 1% worldwide and 0.58% in Nepal.^2^, ^3^ Classical cutaneous lesions of LP are pruritic, and polygonal violaceous flat‐topped papules.^4^

Metabolic Syndrome is a constellation of interrelated metabolic risk factors that appear to directly promote the development of Type 2 Diabetes Mellitus and Cardiovascular Disease.^5^ Skin serves as a mirror of underlying metabolic dysfunction. Recent evidence suggests that chronic inflammation and oxidative stress contribute to a higher risk for Metabolic Syndrome in dermatological disorders.^6^ In 1963, Grinspan reported the association of oral LP with Diabetes Mellitus and Hypertension.^7^

Early detection of Metabolic Syndrome in LP patients helps in predicting cardiovascular risk factors, Diabetes Mellitus, stroke, and colorectal cancer and taking preventive measures as well.^8^ Hence, a prospective hospital‐based case‐control study was conducted to further enlighten this area.

## 3 OBJECTIVE

To study the association of Metabolic Syndrome in patients with LP.

## 4 MATERIAL AND METHODS

Considering the prevalence rate of LP in Nepal to be 0.58%, the minimum sample size (Z score 1.96 for 95% confidence and margin of error, *d* = 3%) was calculated to be 25 however, 75 clinically and histopathologically confirmed adult patients with LP age ≥18 years and 82 age‐sex matched controls were recruited by consultant dermatologists in this hospital‐based prospective study after informed consent. The study was conducted from 5 April 2021 to 31 January 2023. Cases and controls were recruited during the same time frame and from the same hospital. Patients with nevi, verruca vulgaris, molluscum contagiosum, and seborrhoeic keratosis were mainly included in the control group.

Smoking, chronic alcoholism, Diabetes Mellitus, Hypertension, oral corticosteroids, dyslipidemia, and thyroid dysfunction are independently associated with metabolic syndrome and might lead to confounding bias. Thus, patients with these risk factors were excluded from the study. Exclusion criteria were similar for cases and controls. The ratio of the number of cases to controls was 1:1.1 in our study. The study obtained ethics approval from the Institution Review Committee.

Demographic and clinical data were recorded in a proforma. The severity of the disease was assessed using Body Surface Area involvement that is, Mild (1%–3% BSA), Moderate (3%–10% BSA), and Severe (>10% BSA).^9^ Waist Circumference, Systolic, and Diastolic Blood Pressure (DBP) were measured in the study population. Blood samples were taken from participants after 12 h of fasting. Analysis of all samples was performed at the laboratory of the hospital using AGAPPE MISPA CX4 technology.

Metabolic Syndrome was diagnosed according to Revised National Cholesterol Education Programme Adult Treatment Panel III criteria for which at least three of the following criteria is required.^10^

- Abdominal obesity (Waist Circumference ≥90 cm for Asian men or ≥80 cm for Asian women),
- Dyslipidemia (Triglycerides ≥150 mg/dl, High Density Lipoprotein (HDL) cholesterol ≤40 mg/dl for men or ≤50 mg/dl for women)
- Systolic/DBP ≥130/85 mmHg, and
- Fasting Plasma Glucose ≥100 mg/dl

Statistical analysis of the data was performed using Statistical Package for the Social Sciences software, version 26. Means and SDs were calculated for continuous variables and frequency and percentages for categorical ones. The chi‐square test was used for data analysis considering *p* value < 0.05 to be significant.

## 5 RESULTS

A total of 75 cases and 82 controls were included in our study. The age group 31–40 years (30.6%) was predominant. 69.3% were females (2.21:1 female: male ratio) with a mean age of 46.13 ± 14.9 years in cases and 47.04 ± 15.6 years in controls.

**FIGURE 1:**
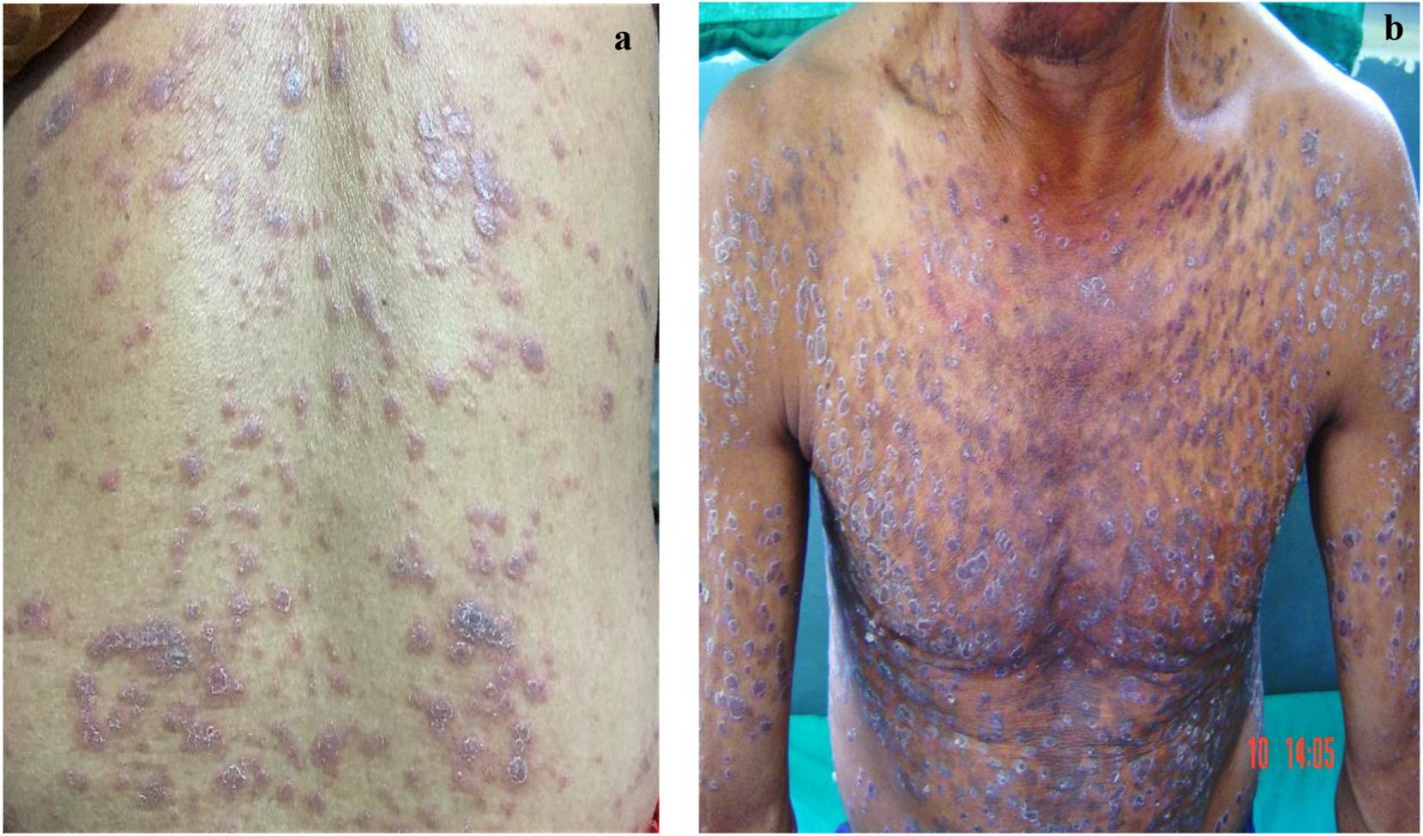
Shows extensive Lichen Planus (LP) in (a) a female (38 years) and (b) male (36 years) who had features of Metabolic Syndrome.

Classical cutaneous LP was observed in the majority of cases (54.6%) as depicted in Figures 1a and 1b followed by only mucosal type (17.3%). The hypertrophic variant was seen in 12% of patients whereas Lichen Planopilaris and LP Pigmentosus were evident in 6.6% each. Annular LP was the least common morphology noted in 2.6% of patients. Mucosal lesions were noted in 26.6% of patients with oral and genital mucosa involvement in 17.3% and 9.3% respectively. 13.3% had nail changes among which 8% had longitudinal ridging, 4% had thinning of nail plates, and 1 patient had pterygium. The majority of the patients (64%) included in our study had a disease duration of 1–6 months. The mean duration of the disease was 14.03 months. Lichen Planus was predominantly moderate in severity (44%) in our study.

The prevalence of Metabolic Syndrome was found to be 32% in patients with LP versus 13.4% in the control group which was statistically significant (*p* 0.005, OR 3.037, 95% CI 1.366–6.754) as depicted in Table 1. Among patients with metabolic syndrome, the majority belonged to the age group between 31 and 40 years (37.5%, *p* 0.378). Metabolic syndrome was found predominantly in females. 71% of Metabolic Syndrome was observed in the females however, it was statistically insignificant (*p* = 0.847). Metabolic syndrome was significantly associated with the duration, severity, and morphology of LP as shown in Table 2.

**TABLE 1:** Distribution of Metabolic Syndrome and Dyslipidemia in cases and controls.

Parameters	Cases (*n* = 75)	Controls (*n* = 82)	*p*‐value	OR	95% CI
Metabolic syndrome	32%	13.4%	0.005*	3.307	1.36–6.75
Dyslipidemia	60%	34%	0.001*	2.893	1.51–5.53

*p value < 0.05 ‐ statistically significant.

Abbreviations: CI, Confidence Interval; OR, Odds Ratio.

**TABLE 2:** Association of duration, severity, and morphology of Lichen Planus (LP) with Metabolic Syndrome.

Parameters	Lichen planus (*n* = 75)		*p*‐value	OR	95% CI
	Metabolic syndrome present (*n*1 = 24)	Metabolic syndrome absent (*n*2 = 51)			
Duration of lichen planus					
≥6 months	15 (55.5%)	12 (44.4%)	0.001*	5.42	1.90–15.47
<6 months	9 (18.7%)	39 (81.2%)			
Severity of lichen planus					
Severe	17 (58.6%)	12 (41.4%)	<0.001*	7.79	2.38–26.47
Moderate	5 (15.1%)	28 (84.9%)			
Mild (ref.)	2 (15.3%)	11 (84.7%)			
Morphology of lichen planus					
Only oral mucosal	8 (61.5%)	5 (38.4%)	0.027*	3.9	1.11–13.75
Classic cutaneous	13 (31.7%)	28 (68.3%)			
Hypertrophic	2 (22.2%)	7 (77.7%)			
Lichen planopilaris	1 (20%)	4 (80%)			

*p < 0.05 ‐ statistically significant.

Abbreviations: CI, Confidence Interval; OR, Odds Ratio.

The prevalence of dyslipidemia was found to be 60% in patients with LP versus 34% in the control group (*p* 0.01, OR 2.893, 95% CI 1.511–5.538) as depicted in Table 1. The components of metabolic syndrome and dyslipidemia for patients and controls have been compared in Table 3.

**TABLE 3:** Comparison of parameters of Metabolic Syndrome in patients with Lichen Planus (LP) and controls.

Parameters	Cases (*n* = 75)	Controls (*n* = 82)	*p*‐value	OR	95% CI
Waist circumference males (≥90 cm)	8 (34.7%)	18 (48.6%)	0.291	0.56	0.19–1.65
Waist circumference females (≥80 cm)	23 (44.2%)	31 (62.2%)	0.076	0.48	0.21–1.09
SBP (≥130 mm of Hg)	38 (50.6%)	28 (34.1%)	0.036*	1.98	1.04–3.77
DBP (≥85 mm of Hg)	24 (32%)	15 (18.2%)	0.047*	2.1	1.00–4.41
Triglycerides (≥150 mg/dl)	35 (40%)	19 (23.1%)	0.002*	2.901	1.46–5.75
HDL (<40 mg/dl)	16 (21.3%)	8 (9.75%)	0.044*	2.508	1.00–6.26
HDL males (≤40 mg/dl)	6 (26.0%)	6 (16.2%)	0.352	1.82	0.51–6.54
HDL females (≤50 mg/dl)	18 (34.6%)	18 (40%)	0.584	0.79	0.35–1.81
Low‐density lipoprotein (>130 mg/dl)	28 (37.3%)	16 (19.5%)	0.013*	2.457	1.20–5.04
Total cholesterol (>200 mg/dl)	28 (37.3%)	19 (23.1%)	0.053	1.98	0.99–3.96
Very low‐density lipoprotein (>30 mg/dl)	14 (18.6%)	9 (10.9%)	0.173	1.862	0.75–4.60
Fasting blood glucose (≥100 mg/dl)	34 (45.3%)	29 (35.3%)	0.203	1.52	0.80–2.88

*p < 0.05 ‐ statistically significant.

Abbreviations: CI, Confidence Interval; DBP, Diastolic Blood Pressure; HDL, High Density Lipoprotein; OR, Odds Ratio; SBP, Systolic Blood Pressure.

## 6 DISCUSSION

Metabolic Syndrome has been reported in around 3%–59% of patients with LP as reported in the literature and was found to be 32% in our study which is in accordance with studies reported previously.^6^ ^,^ ^11^, ^12^, ^13^, ^14^, ^15^ The etiopathogenesis of Metabolic Syndrome in LP is elusive however, the theory of autoimmunity has been proposed.^16^

Interleukins (IL‐1, IL‐6, IL‐9, IL‐23), TNF‐α, and IFN‐γ are reported to be overexpressed in lesions of LP.^17^ TNF‐α decreases insulin sensitivity via inactivation of the Peroxisome Proliferator activated receptor. IL‐6 increases C‐Reactive Protein in the liver which inhibits prostacyclin synthase and induces plasminogen activator inhibitor‐1.^18^, ^19^, ^20^ Similarly, Reactive Oxygen Species cause lipid peroxidation and activate vascular endothelial cells predisposing to atherosclerosis.^21^

Our study showed a significant association of Metabolic Syndrome in patients with LP as reported previously.^12^, ^13^, ^15^, ^22^ Among patients with Metabolic Syndrome, the majority were females which is in accordance with a study by Saxena et al.^15^ The gender difference might be due to the predominant female population in our study. Metabolic Syndrome was predominant in the 4^th^ decade in our study however, Kurian et al, and Rana et al reported metabolic syndrome predominantly in the 5^th^ and 6^th^ decade respectively.^22^, ^23^ The difference might be due to the predominant population of the 31–40 years age group in our study.

Metabolic Syndrome was significantly associated with the duration of LP in our study as observed by Baykal et al which might be due to the prolonged inflammation.^24^ Similarly, the severity of the disease also had a significant association with Metabolic Syndrome as recorded by Daye et al.^25^ This situation is thought to be due to the effect of cytokines increasing due to the increase of disease severity.

Patients with only oral mucosal involvement were also significantly associated with Metabolic Syndrome as reported by Baykal et al.^24^ It has been stated in the literature that oral LP was more common in patients with bad eating habits that is, diets rich in animal fats (e.g red meat, processed and fried food). A permanent high‐fat diet promotes hyperinsulinemia, insulin resistance, dyslipidemia, and increased production of oxidative stress which might lead to Metabolic Syndrome in these patients.^25^, ^26^

Dyslipidemia has been reported in around 35.9%–81.4% of patients with LP as reported in the literature which is due to cytokine‐mediated lipolysis.^6^, ^11^, ^12^ The prevalence of dyslipidemia in our study was 65% which is similar to Shingla et al.^26^ In our study, dyslipidemia was significantly associated with LP which is in accordance with studies reported previously.^12^, ^25^, ^27^

Various patterns of dyslipidemia have been reported in patients with LP. In our study, Serum Triglyceride levels and Low Density Lipoprotein were significantly raised whereas Serum HDL was significantly low in patients with LP. These findings are in accordance with studies reported in the literature.^12^, ^23^, ^27^ Total cholesterol was predominantly recorded in cases however, it was insignificant. Kuntoji et al and Shingla et al showed significant elevation of Total Cholesterol in patients with LP.^12^, ^27^ Though Serum Very Low Density Lipoprotein (VLDL) was elevated in our patients, it was insignificant however, Shingla et al observed significantly raised VLDL in patients with LP.^27^

The development of hypertension in patients with LP has been proposed to be due to decreased prostacyclin synthesis, which is a potent vasodilator. As observed in previous studies, Systolic and DBP were significantly elevated in our patients as well.^22^, ^25^, ^27^

Waist circumference is an indicator of intra‐abdominal adipose tissue. Elevated Waist circumference confers an increased risk of cardiometabolic disease. In our study, waist circumference was not significantly associated with LP which shows metabolic syndrome is multifactorial and does not depend solely on obesity however, Kuntoji et al showed significantly elevated waist circumference in females.^12^

Cytokines‐mediated insulin resistance and lipolysis play a role in hyperglycemia. Fasting blood sugar was not significantly elevated in patients with LP which is similar to the findings recorded by Kurian et al and Kuntoji et al^12^, ^22^ however, Rana et al and Shingla et al showed significantly elevated values in LP patients.^23^, ^27^

Recent evidence suggests that the thickness of the carotid intima‐media and the epicardial fat tissue is significantly increased in patients with LP which proves that patients with LP are prone to develop atherosclerosis.^28^ However, the above findings were beyond the scope of our study.

## 7 CONCLUSION

Our study showed a significant association between LP and Metabolic Syndrome. Metabolic Syndrome was significantly associated with morphology (only oral mucosal involvement, *p* 0.027, OR 3.9), severity (severe, *p* < 0.001, OR 7.93), and duration (≥6 months, *p* 0.001, OR 5.42) of the disease in our study. Thus it is worthwhile to screen for Metabolic Syndrome in patients with LP. This will help in taking preventive measures and early therapeutic intervention in patients having risk factors for Diabetes Mellitus, Cardiovascular diseases, stroke, and colorectal cancer.

## 8 LIMITATIONS

As the study was a single centre based with a smaller sample size, multicentric studies with a larger sample size would be required to draw an inference in the general population.

## CONFLICT OF INTEREST STATEMENT

None to declare.

## AUTHOR CONTRIBUTIONS

**Mahesh Mathur**: Conceptualization (lead); Data curation (lead); Formal analysis (lead); Methodology (lead); Supervision (lead); Visualization (lead); Writing – original draft (lead); Writing – review & editing (lead). **Neha Thakur**: Conceptualization (lead); Data curation (lead); Formal analysis (equal); Methodology (equal); Validation (lead); Visualization (lead); Writing – review & editing (equal). **Sunil Jaiswal**: Formal analysis (equal); Methodology (equal); Validation (lead); Visualization (equal); Writing – original draft (lead); Writing – review & editing (lead). **Gautam Das**: Conceptualization (lead); Data curation (equal); Formal analysis (equal); Methodology (equal); Validation (lead); Visualization (lead). **Swati Shah**: Data curation (lead); Formal analysis (lead); Methodology (equal); Supervision (equal); Validation (lead); Visualization (equal); Writing – original draft (lead). **Srijana Maharjan**: Data curation (equal); Formal analysis (supporting); Methodology (supporting); Validation (equal); Visualization (equal). **Supriya Paudel**: Data curation (supporting); Formal analysis (supporting); Methodology (supporting); Validation (lead); Visualization (equal). **Anjali Shrestha**: Data curation (supporting); Formal analysis (supporting); Investigation (lead); Validation (equal); Visualization (equal). **Hari Prasad Upadhyay**: Formal analysis (lead); Methodology (lead); Supervision (supporting); Validation (lead); Visualization (equal); Writing – original draft (supporting); Writing – review & editing (supporting).

## ETHICS STATEMENT

The study obtained ethics approval from the College of Medical Sciences and Teaching Hospital. Institution Review Committee and all the participants gave informed consent before taking part.

## Data Availability

The data that support the findings of this study are openly available at http://doi.org/10.1002/ski2.315, reference number SKI2315.
